# Sex and Age Differences in Electrocardiographic Parameters Among Adults With Normal ECGs at a Single Diagnostic Centre in Owerri, Nigeria: A Retrospective Cross‐Sectional Study

**DOI:** 10.1111/anec.70257

**Published:** 2026-11-04

**Authors:** Jovita Eghe Egharevba, Clinton Nwanaga Uche, Chosen Nwaeze Egwim, Acomo Grace, Gerald Olwit, Ivaan Pitua, James Nelson Okema

**Affiliations:** ^1^ Department of Physiology, Faculty of Basic Medical Sciences Federal University of Technology Owerri Nigeria; ^2^ Department of Human Physiology, Faculty of Basic Medical Sciences University of Port Harcourt Choba Rivers State Nigeria; ^3^ College of Medicine, Imo State University Owerri Imo State Nigeria; ^4^ Department of Internal Medicine St. Mary's Hospital, Lacor Gulu Uganda; ^5^ Department of Internal Medicine Kabale University School of Medicine Kabale Uganda; ^6^ School of Medicine, College of Health Sciences Makerere University Kampala Uganda; ^7^ Faculty of Medicine Gulu University Gulu Uganda

**Keywords:** aging, electrocardiography, Nigeria, QTc interval, reference values, selection bias, sex differences, sub‐Saharan Africa

## Abstract

**Background:**

Electrocardiographic (ECG) reference values used in Nigerian diagnostic centres are derived from European and North American cohorts, and age‐ and sex‐stratified Nigerian data are scarce.

**Methods:**

In this retrospective cross‐sectional study, 313 12‐lead ECGs reported as normal by a cardiologist at a diagnostic centre in Owerri, Nigeria, were grouped by age (18–40 years, *n* = 164; ≥ 60 years, *n* = 149) and sex. Heart rate, P‐wave duration, PR interval, QRS duration and machine‐reported QTc were compared using *t*‐tests or Mann–Whitney *U* tests and two‐way ANOVA, with Bonferroni correction. Sensitivity analyses applied conventional limits uniformly.

**Results:**

Heart rate, P‐wave duration, PR interval and QRS duration did not differ between age groups (all *p* > 0.09). QTc was longer in older adults (433.7 ± 17.4 vs. 427.4 ± 18.3 ms; *p* = 0.008), also after heart‐rate adjustment (8.3 ms; 95% CI 4.8 to 11.8). Females had longer QTc than males in both age groups (by 14.6 and 11.6 ms; both *p* < 0.001) in all analyses. Males had wider QRS than females only in younger adults (95.7 vs. 89.5 ms; *p* < 0.001), but this age‐by‐sex interaction (*p* = 0.007) was not significant when limits were applied uniformly (*p* = 0.076). PR stopped at 200 ms in both age groups, consistent with selection.

**Conclusion:**

The female‐longer QTc was the most consistent finding, and older adults had modestly longer QTc. Age‐related conduction differences may have been masked by selection; unselected multicentre data are needed before reference intervals are adopted.

## Graphical abstract

In 313 normal 12‐lead ECGs from Nigerian adults, sex not age was the stronger determinant of cardiac conduction: QRS narrowed with age in men but not women, and QTc was longer in women at both ages. Ceiling truncation of PR/QRS values calls for sex‐specific reference limits.

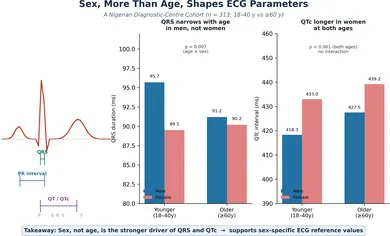

## 1 Introduction

The 12‐lead electrocardiogram (ECG) remains the most widely available, noninvasive tool for evaluating cardiac rhythm, conduction and repolarization, and its interpretation depends fundamentally on comparison against normal reference ranges (Rijnbeek et al. 2014; van der Ende et al. 2017). A large body of evidence shows that these ranges are not fixed across populations: age, sex, and ethnicity each shift the distribution of heart rate, P‐wave duration, PR interval, QRS duration and the corrected QT (QTc) interval (Rijnbeek et al. 2014; van der Ende et al. 2017; Katibi et al. 2013; Sheikh et al. 2014). Reference intervals derived from European cohorts, such as the normative dataset of Rijnbeek et al. for adults aged 16–90 years and the Netherlands‐based LifeLines cohort, are therefore not automatically transferable to African populations (Rijnbeek et al. 2014; van der Ende et al. 2017).

At population level, advancing age is associated with modest prolongation of conduction intervals and with changes in repolarization, even in the absence of overt cardiac disease (Rijnbeek et al. 2014; van der Ende et al. 2017). Distinguishing this age‐associated difference from early pathology requires age‐appropriate reference ranges; without them, benign age‐related change risks being mislabelled as conduction disease, while genuine disease risks being dismissed as age‐related. This study compares two discrete age bands (18–40 and ≥ 60 years) rather than a continuous age range; this design can characterise a between‐group contrast but cannot establish a within‐individual aging trajectory (see Limitations), a distinction we return to throughout.

Sub‐Saharan African data on this question remain limited relative to the size of the population they would serve. Existing studies, including a large Nigerian study of apparently healthy adults that reported normal limits by age and sex, a study of young adults of Yoruba ethnicity, and a rural Tanzanian cohort of adults aged 70 years and older, provide population‐specific normal limits and show sex differences (wider QRS in men, longer QTc in women) that parallel those reported internationally (Katibi et al. 2013; Awoyinka and Ogunlade 2024; Dewhurst et al. 2014). However, few studies have directly compared a defined younger adult group with a defined older adult group drawn from the same diagnostic setting, using tracings that had already been reported as normal by a cardiologist, which is the comparison most relevant to a diagnostic centre deciding which reference range to apply to a given patient.

Owerri, Imo State, is one such setting: to our knowledge no locally derived, age‐stratified ECG dataset previously existed for its diagnostic centres, and clinicians there currently apply reference ranges developed elsewhere. This study addresses that gap using archived ECGs reported as normal at a single diagnostic centre, and examines whether that eligibility process may have introduced selection bias into the resulting values. Because QTc is intended to be independent of heart rate, and the widely used Bazett formula over‐corrects at higher heart rates so that QTc remains positively related to heart rate (Vandenberk et al. 2016; Rautaharju et al. 2009), we also examined how completely the rate correction applied in routine practice removed the effect of heart rate.

### 1.1 Objectives

This study aimed to: (1) compare heart rate, P‐wave duration, PR interval, QRS duration and QTc interval between younger (18–40 years) and older (≥ 60 years) adults with normal ECG tracings; (2) determine whether sex differences in these parameters differ between the two age groups (i.e., test for an age‐by‐sex interaction); (3) characterize the relationship between heart rate and QTc within each age‐and‐sex subgroup, as an assessment of residual rate dependence of the reported QTc; and (4) derive candidate age‐ and sex‐specific descriptive values for this population and compare them with previously published Nigerian and sub‐Saharan African normative data. Two further, non‐prespecified analyses were undertaken in response to the primary results and are reported as exploratory: an examination of PR‐interval and QRS‐duration values near the conventional upper limits, to assess possible truncation by the study's own eligibility criterion, and an indirect evaluation of the QTc correction formula used in the source records.

## 2 Methods

### 2.1 Study Design and Setting

This was a retrospective, comparative, cross‐sectional study conducted using archived electrocardiographic records from a diagnostic centre in Owerri, Imo State, Nigeria. All tracings had been acquired on a single, properly grounded 12‐lead ECG machine (SE‐1200 Express, Edan Instruments, Shenzhen, China) under standardized recording conditions (controlled room temperature, minimized electromagnetic interference and mechanical artifact).

### 2.2 Study Population and Eligibility

Eligible tracings were those from adults whose whole 12‐lead ECG had been reported as normal by the consultant cardiologist at the time of recording. In routine practice at the centre, the cardiologist reviewed each tracing together with the device's automated measurements and interpretation and issued a normal or abnormal report; no study‐specific written criteria were applied, and the numerical limits used in routine reporting were not recorded in the archive. Because the limits may therefore not have been applied identically to every tracing, the limits actually observed in the data are reported for each parameter (Section 3.6). Morphological findings such as early repolarization, anterior T‐wave inversion and isolated QRS voltage criteria for left ventricular hypertrophy were not extracted, and the reasons for individual classifications were not recorded, so whether these patterns led to exclusion could not be determined retrospectively. Each ECG was classified by a single consultant cardiologist, and the normal/abnormal classification itself was not re‐adjudicated for this study. A cardiac physiologist re‐measured the key intervals as a verification step; the physiologist was not blinded to age and sex, which were recorded on the same proforma. Participants were stratified into a younger group (18–40 years) and an older group (≥ 60 years), consistent with the World Health Organization convention for defining older adulthood; the younger band was chosen to represent adults with minimal expected age‐related conduction change. Tracings with artifact were excluded. Exclusion for pathology was based on the cardiologist's ECG report alone; clinical histories, comorbidity and medication records were not reviewed, so the absence of cardiovascular disease could not be confirmed.

The structured proforma recorded age, sex and the ECG parameters only; nationality, place of birth, ethnicity, language and the reason for referral were not extracted and could not be reconstructed retrospectively from the de‐identified archive. The centre serves patients from Owerri and its environs in south‐eastern Nigeria, but residence in this catchment area does not establish Nigerian nationality or ethnic ancestry. The sample is therefore described throughout as adults attending a single diagnostic centre in Owerri, and comparisons with cohorts of documented ethnicity are not interpreted as ethnic comparisons. Habitual physical activity was not recorded.

### 2.3 Sampling and Sample Size

Purposive sampling was used to identify all tracings meeting the age and “normal” classification criteria in the centre's archive, from the centre's inception to the date of data selection. The final analytic sample comprised 313 individuals (164 younger, 149 older), exceeding the minimum of 70–100 tracings anticipated at the proposal stage. No formal a priori power calculation was performed. For descriptive limits, the CLSI EP28‐A3c guideline recommends at least 120 reference individuals per partition for nonparametric estimation of the 2.5th and 97.5th percentiles (Clinical and Laboratory Standards Institute 2010); the four age‐by‐sex partitions here contained 62 to 102 individuals, so their percentile limits are imprecise and should be interpreted with caution.

### 2.4 Variables and Data Extraction

For each tracing, the following variables were extracted onto a structured proforma: sex, age, heart rate (bpm), P‐wave duration (ms), PR interval (ms), QRS duration (ms), and QTc interval (ms). All values were taken from the cardiologist's report, which served as the primary reference; the interval values in these reports were the device's automated measurements as reviewed by the cardiologist. Consistent with this, all P‐wave, PR‐interval and QRS‐duration values in the dataset were recorded at 2‐ms resolution (even integers). The cardiac physiologist's re‐measurement was used to verify these values, and the reported value was retained in the dataset in all cases. Automated systems measure global intervals from the earliest onset to the latest offset across simultaneously recorded leads, which are systematically longer than single‐lead measurements (Kligfield et al. 2007). QTc was the value reported by the ECG machine. The SE‐1200 Express can report QTc using several correction formulae (Bazett, Fridericia, Framingham and Hodges); the formula configured at the centre was not documented in the archived reports and could not be confirmed retrospectively, so QTc is described throughout as the machine‐reported value. Formal inter‐rater agreement statistics were not computed. The uncorrected QT interval, the RR interval and voltage parameters for left ventricular hypertrophy (e.g., Cornell and Sokolow–Lyon criteria) were not extracted, consistent with the study's restriction to basic conduction and repolarization parameters.

### 2.5 Statistical Analysis

All analyses were performed on the individual‐level dataset in Python 3.13 (pandas 3.0.5, NumPy 2.5.3, SciPy 1.18.1, statsmodels 0.15.0). Categorical variables (age group, sex) were summarized as frequencies and percentages and compared using the chi‐square test. Continuous variables were expressed as mean ± standard deviation, and their distribution was assessed with the Shapiro–Wilk test. Between‐group comparisons used Welch's *t*‐test for approximately normally distributed variables and the Mann–Whitney *U* test otherwise; the test used is reported for each comparison. Mean differences, their 95% confidence intervals (Welch) and Cohen's *d* (pooled standard deviation) were calculated from the individual‐level data. Sex differences were examined separately within each age group, and a two‐way ANOVA (parameter~age group × sex, type II sums of squares) was fitted for each parameter to test the age‐by‐sex interaction, that is, whether the male–female difference differed between age groups. Because Shapiro–Wilk testing indicated non‐normality in at least one subgroup for every parameter, each interaction was also tested with the aligned rank transform (ART) ANOVA, a nonparametric procedure for factorial designs (Wobbrock et al. 2011), with heteroscedasticity‐consistent (HC3) standard errors, and, for QRS duration and QTc, with a permutation test (5000 permutations of residuals from the main‐effects model). The size of each interaction was expressed as a difference in differences with a 95% bootstrap confidence interval (5000 resamples within each age‐by‐sex cell). The relationship between heart rate and QTc was assessed within each age‐and‐sex subgroup using Pearson correlation and linear regression, with Spearman correlation as a nonparametric check, and the age and sex differences in QTc were re‐estimated with heart rate as a covariate (ANCOVA).

Because five ECG parameters were tested within each comparison family (the age‐group family in Table 2, the sex‐within‐age‐group family in Table 3, and the interaction family in Table 4), a Bonferroni‐corrected threshold of *α* = 0.01 (0.05/5) was applied as the primary criterion for statistical significance within each family, in addition to the nominal *α* = 0.05; both are reported. Effect sizes (Cohen's *d*) are reported as an approximate, standardized index of magnitude alongside the test result; *d* assumes approximate normality and should be read as indicative where Mann–Whitney *U* was used. Three groups of exploratory analyses were undertaken after the primary analyses and are labeled as post hoc and hypothesis‐generating: (i) an examination of the observed range of each parameter and the number of tracings outside conventional limits (Section 3.6); (ii) an evaluation of the residual relationship between heart rate and QTc (Section 3.7); and (iii) sensitivity analyses repeating the main comparisons after applying conventional limits uniformly to all tracings, first for heart rate (60–100 bpm), PR interval (120–200 ms) and QRS duration (≤ 110 ms), and then additionally excluding QTc values at or above the sex‐specific prolongation thresholds (450 ms in men, 460 ms in women) (Rautaharju et al. 2009; Surawicz et al. 2009), together with Spearman correlations between age and each interval within the older group (Section 3.8).

### 2.6 Ethical Considerations

Ethical approval for this study was obtained from the School of Basic Medical Sciences Ethics and Research Committee, Federal University of Technology Owerri (FUTO), Nigeria (Approval No. FUT/COM/SBMS/REC/Vol1/002; approved on 27 October 2025). All data were extracted retrospectively from the archive after ethical approval had been obtained. The study was conducted in accordance with the ethical principles of the Declaration of Helsinki. This manuscript was prepared with reference to the STROBE (Strengthening the Reporting of Observational Studies in Epidemiology) statement for cross‐sectional studies.

The study utilized existing data collected during routine institutional activities. All data were anonymized prior to extraction and analysis. No personal identifiers, including participant names, hospital numbers, or other identifying information, were collected. As the study involved retrospective analysis of fully de‐identified data without direct participant contact or intervention, the research posed minimal risk to participants. The data were used exclusively for academic and research purposes, and strict confidentiality was maintained throughout the study.

## 3 Results

### 3.1 Study Population

Of 313 adults with normal ECG tracings, 164 (52.4%) were in the younger group (18–40 years; mean age 31.5 ± 5.9 years) and 149 (47.6%) in the older group (≥ 60 years; mean age 68.8 ± 6.9 years, range 60–90). Overall, 132 participants (42.2%) were male and 181 (57.8%) females; the sex distribution did not differ significantly between age groups (*χ* ^2^ = 2.33, *p* = 0.127; Table 1). Because the proportion of males was numerically higher in the older group (47.0% vs. 37.8%), age‐group comparisons were also made separately within each sex (Section 3.3).

**TABLE 1:** Demographic characteristics of the study population.

Characteristic	Younger adults (18–40 year) *n* = 164	Older adults (≥ 60 year) *n* = 149	Total *N* = 313
Age, years (mean ± SD)	31.5 ± 5.9	68.8 ± 6.9	—
Age range, years	19–40	60–90	19–90
Male, *n* (%)	62 (37.8)	70 (47.0)	132 (42.2)
Female, *n* (%)	102 (62.2)	79 (53.0)	181 (57.8)

*Note:* Sex distribution did not differ significantly between age groups (*χ* ^2^ = 2.33, df = 1, *p* = 0.127).

### 3.2 Age‐Group Comparison of ECG Parameters

Heart rate, P‐wave duration, PR interval and QRS duration did not differ significantly between younger and older adults (all *p* > 0.09; Table 2). QTc interval was significantly longer in older adults than younger adults (433.7 ± 17.4 vs. 427.4 ± 18.3 ms; *p* = 0.008), and this remained significant at the Bonferroni‐corrected family‐wise threshold (*α* = 0.01), although the effect size was small‐to‐moderate (Cohen's *d* = −0.35). After adjustment for heart rate and sex, QTc remained 8.3 ms longer in older adults (95% CI 4.8 to 11.8; *p* < 0.001). The distribution of each parameter by age group and sex is shown in Figure 1.

**TABLE 2:** Comparison of ECG parameters between younger and older adults (pooled sexes).

Parameter	Younger (*n* = 164)	Older (*n* = 149)	95% CI of diff (Y − O)	Cohen's *d*	Test used	*p*	Sig. at *α* = 0.01
Heart rate, bpm	78.2 ± 10.0	76.7 ± 10.4	−0.74, 3.81	0.15	Mann–Whitney *U*	0.121	No
P‐wave duration, ms	114.2 ± 9.3	115.0 ± 12.3	−3.28, 1.61	−0.08	Mann–Whitney *U*	0.371	No
PR interval, ms	161.9 ± 17.6	163.0 ± 20.4	−5.39, 3.13	−0.06	Mann–Whitney *U*	0.325	No
QRS duration, ms	91.9 ± 8.7	90.7 ± 8.1	−0.67, 3.06	0.14	Mann–Whitney *U*	0.360	No
QTc interval, ms	427.4 ± 18.3	433.7 ± 17.4	−10.26, −2.33	−0.35	Mann–Whitney *U*	0.008	Yes

*Note:* Shapiro–Wilk testing indicated departure from normality for at least one group for every parameter (*p* < 0.05 in ≥ 1 subgroup); Mann–Whitney *U* was therefore used uniformly for consistency across this table. “Sig. at *α* = 0.01” denotes significance at the Bonferroni‐corrected family‐wise threshold. Cohen's *d* calculated as (Younger − Older)/pooled SD. Mean differences and 95% CIs (Welch) were calculated from individual‐level data and are reported as a supplement to the primary Mann–Whitney *U* result.

**FIGURE 1:**
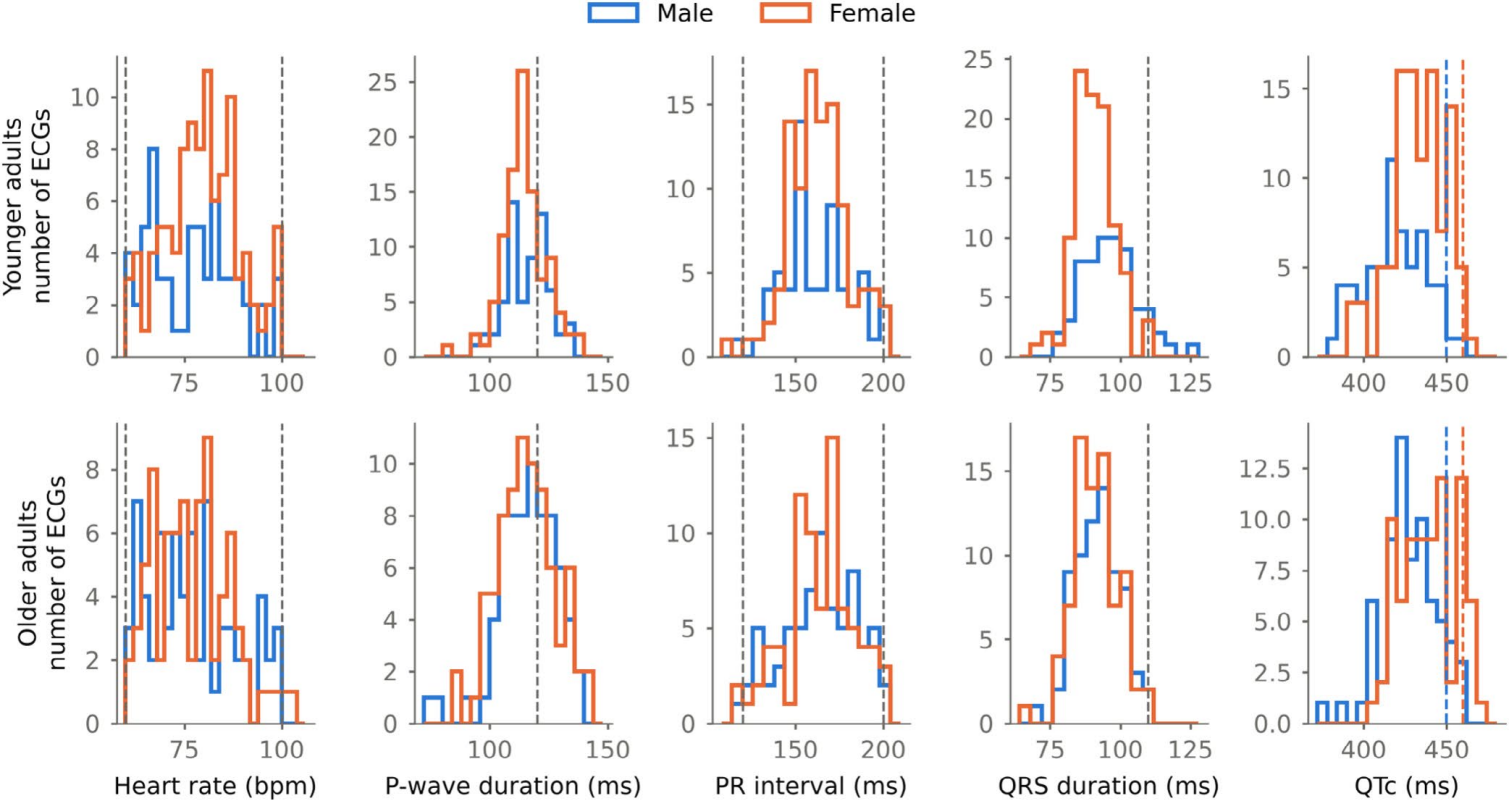
Distribution of each ECG parameter by age group (rows) and sex (blue, male; orange, female), in 2‐ms to 6‐ms bins. Dashed gray lines mark conventional limits (heart rate 60 and 100 bpm; P‐wave duration 120 ms; PR interval 120 and 200 ms; QRS duration 110 ms); dashed blue and orange lines mark the QTc prolongation thresholds for men (450 ms) and women (460 ms). The PR interval stops at 200 ms in both age groups and no older adult has a QRS duration above 110 ms, whereas QTc extends beyond the prolongation thresholds.

Age (as a continuous variable) correlated only weakly with QTc across the whole sample (*r* = 0.19, *p* < 0.001) and not significantly with the other four parameters. This whole‐sample correlation should be read cautiously: because the design deliberately excludes the 41‐ to 59‐year band, the age distribution is bimodal rather than continuous, and a Pearson correlation computed across such a gap can visually overstate the appearance of a smooth linear trend. The two‐group comparison above is the more defensible test of the age effect for this design; the continuous‐age correlation is reported for completeness only.

### 3.3 Sex Differences Within Each Age Group, and the Age‐By‐Sex Interaction

Within the younger group, males had significantly wider QRS complexes than females (95.7 ± 9.5 vs. 89.5 ± 7.3 ms; difference 6.1 ms; *p* < 0.001; *d* = 0.75) and significantly shorter QTc intervals (418.3 ± 18.2 vs. 433.0 ± 16.1 ms; difference 14.6 ms; *p* < 0.001; *d* = −0.87). Heart rate, P‐wave duration and PR interval did not differ significantly by sex in this group (Table 3).

**TABLE 3:** Sex‐stratified comparison of ECG parameters within each age group.

Parameter	Male mean ± SD	Female mean ± SD	95% CI of diff (M − F)	Cohen's *d*	Test used	*p*
Younger adults (*n* = 62 M, 102 F)						
Heart rate, bpm	76.4 ± 10.7	79.3 ± 9.4	−6.15, 0.40	−0.29	Mann–Whitney *U*	0.097
P‐wave duration, ms	115.2 ± 9.0	113.5 ± 9.4	−1.27, 4.55	0.18	Mann–Whitney *U*	0.268
PR interval, ms	161.3 ± 19.0	162.2 ± 16.8	−6.78, 4.82	−0.06	Welch *t*‐test	0.739
QRS duration, ms	95.7 ± 9.5	89.5 ± 7.3	3.35, 8.91	0.75	Welch *t*‐test	< 0.001***
QTc interval, ms	418.3 ± 18.2	433.0 ± 16.1	−20.19, −9.07	−0.87	Mann–Whitney *U*	< 0.001***
Older adults (*n* = 70 M, 79 F)						
Heart rate, bpm	76.6 ± 11.0	76.7 ± 9.8	−3.51, 3.28	−0.01	Mann–Whitney *U*	0.815
P‐wave duration, ms	115.3 ± 12.6	114.7 ± 12.2	−3.44, 4.60	0.05	Mann–Whitney *U*	0.499
PR interval, ms	163.1 ± 21.7	162.9 ± 19.4	−6.57, 6.81	0.01	Welch *t*‐test	0.972
QRS duration, ms	91.2 ± 8.1	90.2 ± 8.1	−1.58, 3.68	0.13	Welch *t*‐test	0.430
QTc interval, ms	427.5 ± 16.8	439.2 ± 16.1	−16.97, −6.30	−0.71	Mann–Whitney *U*	< 0.001***

*Note:* ***p < 0.001. Test selected per comparison using Shapiro–Wilk normality results for the two sex subgroups being compared. Mean differences (Male − Female) and 95% CIs (Welch) were calculated from individual‐level data and may differ by 0.1 ms from differences between the rounded means shown.

Within the older group, the QRS sex difference was not statistically significant (91.2 ± 8.1 ms males vs. 90.2 ± 8.1 ms females; difference 1.1 ms; *p* = 0.43; *d* = 0.13). The QTc sex difference persisted (427.5 ± 16.8 ms males vs. 439.2 ± 16.1 ms females; difference 11.6 ms, compared with 14.6 ms in the younger group; *p* < 0.001; *d* = −0.71). Heart rate, P‐wave duration and PR interval again did not differ significantly by sex (Table 3).

Two‐way ANOVA showed a statistically significant age‐by‐sex interaction for QRS duration (*F* = 7.35, *p* = 0.007), significant at the Bonferroni‐corrected threshold, indicating that the male–female QRS difference was smaller in the older group than in the younger group (difference in differences 5.1 ms, 95% bootstrap CI 1.2 to 8.8). The interaction was also present with the aligned rank transform (*p* = 0.014), HC3 standard errors (*p* = 0.009) and the permutation test (*p* = 0.007), although the aligned rank transform result did not meet the Bonferroni threshold. Because the data are cross‐sectional, this is a difference between age groups and not evidence of change within individuals. No significant interaction was found for QTc (*p* = 0.435), heart rate (*p* = 0.238), P‐wave duration (*p* = 0.670), or PR interval (*p* = 0.802), with consistent results from the alternative methods. For QTc, a nonsignificant interaction does not establish that the sex difference is the same in both age groups: the female‐minus‐male difference was 3.0 ms smaller in older adults, with a 95% bootstrap CI of −4.5 to 10.6 ms that is compatible with no change or with moderate narrowing (Table 4).

**TABLE 4:** Two‐way ANOVA: Main effects of age group and sex, and the age‐by‐sex interaction, for each ECG parameter.

Parameter	Age group *p*‐value	Sex *p*‐value	Age × Sex interaction *p*‐value	Sig. interaction at *α* = 0.01
Heart rate, bpm	0.228	0.193	0.238	No
P‐wave duration, ms	0.552	0.368	0.670	No
PR interval, ms	0.590	0.841	0.802	No
QRS duration, ms	0.100	< 0.001***	0.007**	Yes
QTc interval, ms	< 0.001***	< 0.001***	0.435	No

*Note:* **p < 0.01, ***p < 0.001. Type II sums of squares. The significant Age × Sex interaction for QRS duration indicates that the male–female QRS difference was smaller in the older than in the younger group; the absence of a significant interaction for QTc does not show that the sex difference is identical across age groups (14.6 ms in younger and 11.6 ms in older adults). “Sig. interaction at *α* = 0.01” applies the Bonferroni‐corrected family‐wise threshold to the five interaction tests in this table. Interaction *p*‐values from the aligned rank transform, HC3 standard errors and permutation tests, and differences in differences with bootstrap CIs, are given in Section 3.3.

Because the sex ratio differed numerically between age groups (37.8% male in the younger group vs. 47.0% male in the older group; Table 1), we additionally compared each parameter between age groups separately within males and within females (Welch's *t*‐test), to assess whether this imbalance affected the pooled age comparison in Table 2. Within males, QRS duration was significantly narrower in the older group (91.2 ± 8.1 vs. 95.7 ± 9.5 ms; mean difference −4.5 ms, 95% CI −7.5 to −1.4; *p* = 0.005) and QTc was significantly longer (427.5 ± 16.8 vs. 418.3 ± 18.2 ms; mean difference 9.2 ms, 95% CI 3.2 to 15.3; *p* = 0.003). Within females, the QRS difference was small and nonsignificant (90.2 ± 8.1 vs. 89.5 ± 7.3 ms; *p* = 0.59), while QTc was again longer in older women (439.2 ± 16.1 vs. 433.0 ± 16.1 ms; mean difference 6.2 ms, 95% CI 1.5 to 11.0; *p* = 0.011). The QRS age‐by‐sex interaction is therefore not explained by the differing sex ratios: in this sample, older males had a narrower QRS duration than younger males. This cross‐sectional difference may reflect selection, because excluding ECGs not reported as normal would be expected to remove older adults with age‐related conduction change (Sections 3.6 and 3.8).

### 3.4 Age‐ and Sex‐Specific Descriptive Values, Conditional on Prior Normal Classification

Descriptive statistics and empirical 95% ranges (2.5th–97.5th percentile) for each subgroup are presented in Table 5. These values should be read as descriptive of this cardiologist‐preselected sample, not as unselected population reference intervals: Section 3.6 shows distributions of PR interval and QRS duration that are consistent with truncation at the conventional upper limits by the eligibility criterion, and each partition was smaller than the 120 individuals recommended by CLSI EP28‐A3c for nonparametric limits (Clinical and Laboratory Standards Institute 2010). The values are offered as candidate inputs for a future, prospectively designed, multicentre reference‐interval study using an unselected sampling frame.

**TABLE 5:** Age‐ and sex‐specific descriptive values (mean ± SD, median, 95% empirical range) for ECG parameters in this cardiologist‐preselected sample.

Subgroup	*n*	Mean ± SD	Median	95% range
Heart rate, bpm				
Younger M	62	76.4 ± 10.7	77.0	60–98
Younger F	102	79.3 ± 9.4	79.5	62–98
Older M	70	76.6 ± 11.0	75.5	61–98
Older F	79	76.7 ± 9.8	76.0	62–98
P‐wave duration, ms				
Younger M	62	115.2 ± 9.0	116.0	98–132
Younger F	102	113.5 ± 9.4	114.0	95–133
Older M	70	115.3 ± 12.6	116.0	85–135
Older F	79	114.7 ± 12.2	114.0	94–138
PR interval, ms				
Younger M	62	161.3 ± 19.0	160.0	131–199
Younger F	102	162.2 ± 16.8	162.0	132–197
Older M	70	163.1 ± 21.7	166.0	120–197
Older F	79	162.9 ± 19.4	162.0	120–198
QRS duration, ms				
Younger M	62	95.7 ± 9.5	95.0	80–114
Younger F	102	89.5 ± 7.3	88.0	76–105
Older M	70	91.2 ± 8.1	92.0	78–107
Older F	79	90.2 ± 8.1	90.0	78–104
QTc interval, ms				
Younger M	62	418.3 ± 18.2	419.0	386–449
Younger F	102	433.0 ± 16.1	434.5	396–458
Older M	70	427.5 ± 16.8	426.0	397–457
Older F	79	439.2 ± 16.1	438.0	413–465

*Note:* Empirical 95% ranges are descriptive of this preselected sample and should be interpreted as candidate values pending prospective, multicentre validation with a prespecified reference‐interval methodology (e.g., CLSI EP28‐A3c) in an unselected population.

### 3.5 Heart Rate–QTc Relationship

Heart rate correlated positively with QTc interval in every subgroup, indicating residual rate dependence of the machine‐reported QTc (Figure 2). The association was strongest in younger adults (younger males: *r* = 0.45, *p* < 0.001; younger females: *r* = 0.40, *p* < 0.001) and weaker in older adults (older males: *r* = 0.31, *p* = 0.008; older females: *r* = 0.23, *p* = 0.038), though the corresponding Spearman correlation for older females did not reach significance [*ρ* = 0.18, *p* = 0.103], so this last association should be treated as tentative. The regression slopes ranged from 0.38 to 0.77 ms of QTc per bpm across subgroups and were 0.60 ms per bpm (95% CI 0.41 to 0.78) in the whole sample.

**FIGURE 2:**
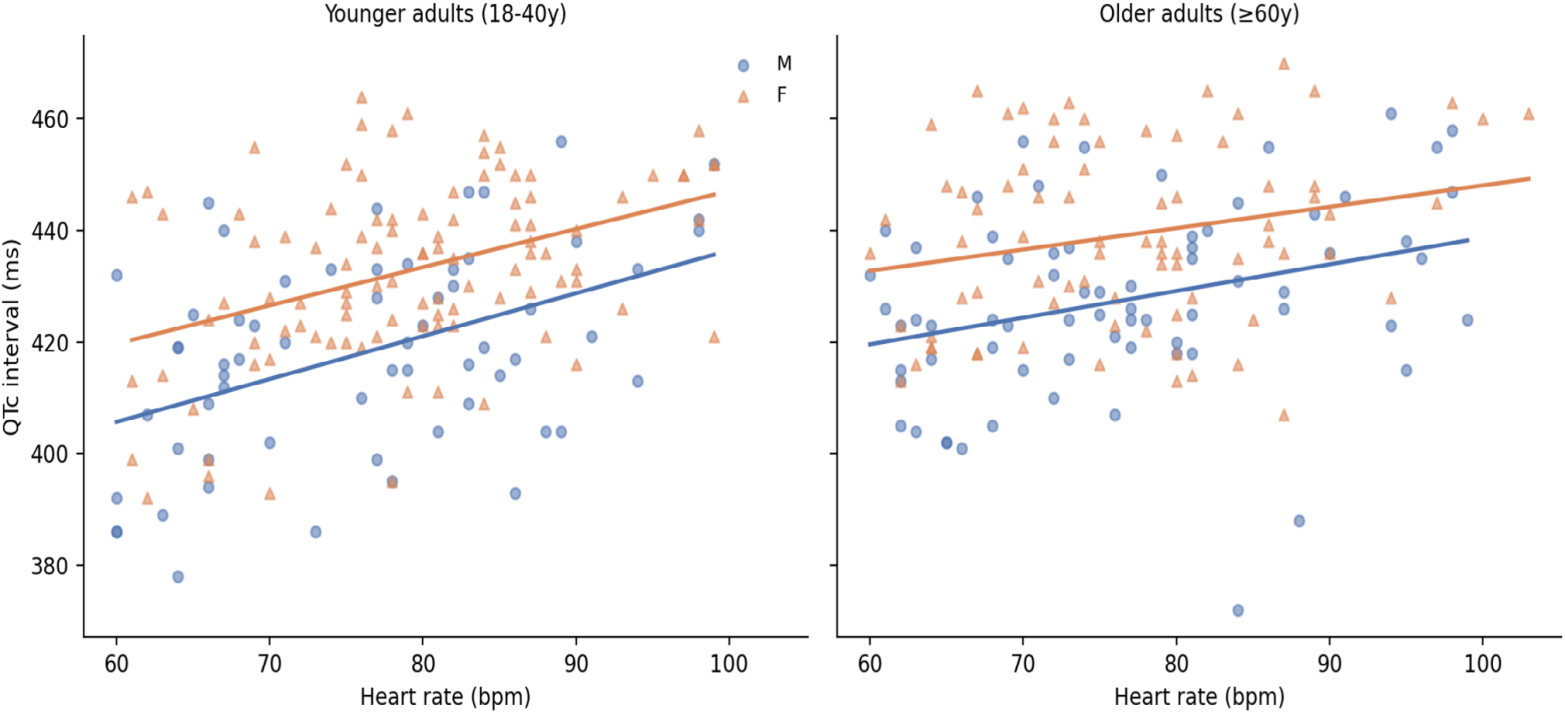
Scatterplots of heart rate versus QTc interval by sex, for younger adults (left) and older adults (right), with linear fit lines. The positive slope in all four subgroups reflects residual rate‐dependence of QTc; the relationship is visually flatter in older adults, consistent with the weaker correlation coefficients reported in Section 3.5.

### 3.6 Observed Ranges and Values Outside Conventional Limits

Because eligibility required a normal ECG report, this post hoc analysis examined the observed range of each parameter and the number of tracings outside conventional limits (Table 6 and Figure 1). In both age groups the maximum PR interval was exactly 200 ms (seven tracings at 200 ms and none above), and 8.5% of younger and 10.7% of older tracings had a PR interval of 190 ms or more (Table 6). An abrupt stop at the cut‐point for first‐degree atrioventricular block, rather than a tapering right tail, is consistent with 200 ms having acted as an exclusion boundary in both age groups. No tracing had a heart rate below 60 bpm, and the minimum was exactly 60 bpm in three of the four subgroups, consistent with sinus bradycardia having been excluded; one older woman had a heart rate of 103 bpm. Five tracings (1.6%) had a PR interval below 120 ms (112–118 ms). No QRS duration in the older group exceeded 110 ms, whereas four younger males had values above the recommended adult limit of 110 ms (Surawicz et al. 2009) (112, 112, 116 and 124 ms); in total, 9 younger (5.5%) and 3 older (2.0%) tracings had a QRS duration of 110 ms or more. These numbers are small, and the larger number of wide QRS values in the younger group partly reflects the wider QRS duration of younger males (Table 3). In contrast, QTc was not truncated at conventional limits: 24 tracings (7.7%) had a QTc at or above the sex‐specific prolongation thresholds (Rautaharju et al. 2009), most often in older women (13 of 79, 16.5%) and older men (7 of 70, 10.0%). P‐wave duration was 120 ms or more in 104 tracings (33.2%; 23.5% to 40.0% across subgroups); because this threshold was derived from single‐lead measurement (Bayés de Luna et al. 2012), whereas automated global measurements are systematically longer (Kligfield et al. 2007), this most likely reflects the measurement method rather than atrial conduction delay. Overall, these data show that the upper limits for PR interval and, in the older group, QRS duration were applied as exclusion boundaries, that other limits were not applied uniformly, and that QTc was not used as an exclusion criterion. They are consistent with selection on conduction intervals, but they do not show that selection explains the absence of an age effect for PR interval or QRS duration.

**TABLE 6:** Proximity of PR interval and QRS duration to conventional abnormal‐conduction thresholds, by age group.

Metric	Younger (*n* = 164)	Older (*n* = 149)	Conventional threshold	Interpretation
PR interval ≥ 190 ms, *n* (%)	14 (8.5%)	16 (10.7%)	> 200 ms = 1° AV block	Both groups stop at exactly 200 ms
Maximum PR interval, ms	200	200	—	Abrupt stop at the cut‐point
QRS duration ≥ 110 ms, *n* (%)	9 (5.5%)	3 (2.0%)	> 110 ms = intraventricular conduction delay (adults)	Small numbers; may partly reflect wider QRS in younger males
Maximum QRS duration, ms	124	110	—	Younger maximum exceeds the adult limit

For ease of comparison, Table 7 below situates the present‐study values alongside two previously published Nigerian and sub‐Saharan African datasets; interpretation of this comparison, including the QTc formula caveat, is taken up in the Discussion.

**TABLE 7:** Comparison of present‐study ECG parameters with two previously published Nigerian and sub‐Saharan African datasets.

Parameter (M/F)	Present study—Younger	Present study—Older	Katibi et al. (2013), Nigeria, age 20–87	Dewhurst et al. (2014), Tanzania, age ≥ 70
PR interval, ms	161/162	163/163	Not available^a^	158/152
QRS duration, ms	95.7/89.5	91.2/90.2	87.9/83.4	89/84
QTc, ms (formula)	418/433 (device‐reported)	428/439 (device‐reported)	393/406 (Hodges)	421/427 (adjusted^b^)

*Note:* Present‐study QTc values are those reported by the ECG machine; the correction formula could not be confirmed. Katibi et al. used the Hodges formula.

Abbreviation: M/F = male/female mean values.

^a^ Mean PR interval not available; in the related thesis the 96th‐percentile range was 110–216 ms in men and 108–224 ms in women, with no age association (Katibi 2011). Cross‐study QTc comparisons are indicative only.

^b^ Values from Dewhurst et al. are adjusted for age, systolic blood pressure, body mass index and RR interval, whereas present‐study values are unadjusted.

### 3.7 Exploratory Analysis of the Residual Rate Dependence of QTc

The source records did not document which rate‐correction formula generated the QTc values, and the uncorrected QT and RR intervals were not extracted. The SE‐1200 Express supports several correction formulae (Bazett, Fridericia, Framingham, and Hodges), so the formula cannot be inferred from the device model. As an exploratory check, we examined the slope of QTc on heart rate. Bazett's formula over‐corrects at higher heart rates and therefore overestimates QTc, producing a positive residual slope, whereas Fridericia's and Framingham's corrections leave less residual rate dependence (Vandenberk et al. 2016). The observed slopes were positive in all four subgroups (0.38–0.77 ms of QTc per bpm; Pearson *p* ≤ 0.038; Section 3.5). This shows that the reported QTc remained dependent on heart rate, but it does not identify the formula used. Because heart rate did not differ significantly between age groups or between sexes within age groups (Tables 2 and 3), and the age and sex differences in QTc were unchanged after adjustment for heart rate (older vs. younger 8.3 ms, 95% CI 4.8 to 11.8; male vs. female −12.3 ms, 95% CI −15.8 to −8.7; interaction *p* = 0.698), residual rate dependence does not explain the QTc findings.

### 3.8 Sensitivity Analyses Applying Conventional Limits Uniformly

Applying the conventional limits for heart rate, PR interval and QRS duration uniformly removed 10 tracings (*n* = 303). PR interval and QRS duration still did not differ between age groups. The QRS sex difference remained significant in younger adults (4.7 ms; *p* < 0.001) and nonsignificant in older adults (1.4 ms; *p* = 0.275), but the age‐by‐sex interaction was no longer significant (*p* = 0.076), indicating that the interaction depended partly on the four younger‐male tracings above 110 ms for which no older counterpart was retained. The QTc findings were unchanged: QTc was longer in older adults (5.6 ms; *p* = 0.024; heart‐rate‐adjusted 8.3 ms, 95% CI 4.6 to 11.9) and longer in females in both age groups (14.4 and 11.6 ms; both *p* < 0.001). When QTc values at or above the sex‐specific prolongation thresholds were also excluded (*n* = 280), the female‐longer QTc persisted in both age groups (15.1 and 10.3 ms; both *p* < 0.001). The unadjusted age difference in QTc was attenuated (2.3 ms; *p* = 0.437), as expected when the upper tail is removed predominantly from older adults, but remained after adjustment for heart rate (5.3 ms, 95% CI 1.8 to 8.9; *p* = 0.003). Within the older group (60–90 years), age was not correlated with PR interval (ρ = 0.06, *p* = 0.44), QRS duration (ρ = −0.10, *p* = 0.23) or QTc (ρ = 0.04, *p* = 0.62).

## 4 Discussion

This study compared ECG parameters between younger and older adults whose ECGs had been reported as normal at a single diagnostic centre in Owerri. Females had a longer QTc than males in both age groups, a finding that was unchanged by adjustment for heart rate and in every sensitivity analysis; older adults had a modestly longer QTc than younger adults; and the male–female difference in QRS duration was smaller in older than in younger adults, although this interaction was not robust. Because inclusion depended on a normal ECG report, which is most likely to distort the age comparison, the sizes of the sex and age effects in this sample cannot be compared to judge which matters more. The paragraphs below consider the null PR and QRS age findings and the role of selection, the QTc correction formula, the QTc and QRS findings, ECG morphology and unmeasured factors, comparison with published African data, and implications.

The absence of a significant age difference in PR interval and QRS duration contrasts with population data showing modest age‐related prolongation of conduction intervals (Rijnbeek et al. 2014; van der Ende et al. 2017). However, an earlier study of apparently healthy Nigerians also found no significant variation in QRS duration across age groups and no age association for the PR interval, and reported 96th‐percentile upper limits for the PR interval of 216 ms in men and 224 ms in women (Katibi 2011). A 200‐ms cut‐off would therefore truncate the PR distribution of healthy Nigerian adults. Selection on a normal ECG is one possible explanation for the null findings. The PR interval stopped at exactly 200 ms in both age groups, consistent with the cut‐point for first‐degree atrioventricular block having acted as an exclusion boundary, and no older adult had a QRS duration above 110 ms, whereas QTc, which was evidently not used as an exclusion criterion, extended beyond its conventional limits. Excluding ECGs not reported as normal would be expected to remove older adults with age‐related conduction change, and could flatten or even reverse an age effect; the absence of any correlation between age and PR or QRS within the older group is compatible with this. However, the numbers near the QRS limit were small, and these data do not show that selection explains the null findings; a true absence of an age‐related difference in this population cannot be excluded. Either way, the descriptive values in Table 5 are conditional on prior normal classification and are not unselected population reference limits.

The correction formula used by the device was not documented in the source records and could not be confirmed retrospectively. The positive residual slope of QTc on heart rate shows that the reported QTc remained rate dependent, a pattern expected with Bazett's formula, which over‐corrects at higher heart rates (Vandenberk et al. 2016; Rautaharju et al. 2009); however, the same finding does not identify the formula, and the device can report QTc using several formulae. The QTc values are therefore interpreted as machine‐reported values, and cross‐study QTc comparisons (Table 7) are treated as indicative. Residual rate dependence did not explain the within‐study QTc differences because heart rate did not differ between the groups compared and the age and sex differences were unchanged after adjustment for heart rate (Section 3.7).

QTc was the only parameter with a statistically significant, though modest, age difference (*d* = −0.35), and this difference persisted after adjustment for heart rate. The QTc distribution was not truncated at conventional limits (Section 3.6), so QTc appears to have been less affected by selection on a normal ECG than PR interval and QRS duration. Females had a longer QTc than males in both age groups (14.6 ms in younger and 11.6 ms in older adults), consistent with the female‐longer QTc reported in European, Nigerian and other sub‐Saharan African cohorts (Rijnbeek et al. 2014; Katibi et al. 2013; Awoyinka and Ogunlade 2024; Dewhurst et al. 2014). The absence of a significant age‐by‐sex interaction does not show that this difference is stable with age: the 95% confidence interval for the change in the sex difference (−4.5 to 10.6 ms) is compatible with moderate narrowing. Some narrowing in older adults would be physiologically plausible, because the adult sex difference largely reflects shortening of the QT interval in males after puberty (Rautaharju et al. 1992) and testosterone shortens ventricular repolarization (Sedlak et al. 2012). The present data are best summarized as showing a sex difference in QTc in both age groups, which supports the established practice of sex‐specific QTc interpretation (Rautaharju et al. 2009).

The significant age‐by‐sex interaction for QRS duration (*p* = 0.007) arose because the male–female difference was 6.1 ms in younger adults but 1.1 ms in older adults, owing to a narrower QRS in older than in younger males. QRS duration tends to increase modestly with age in the general population (van der Ende et al. 2017), so a narrower QRS in older males is unexpected. The sensitivity analysis suggests a methodological explanation: when the conventional QRS limit was applied uniformly, removing four younger‐male tracings above 110 ms that had no older counterpart, the interaction was no longer significant (*p* = 0.076). Older men with age‐related conduction delay may also have been more likely to have an ECG reported as abnormal and excluded. Cohort differences between the two age groups, and unmeasured factors such as body size and habitual physical activity, could also contribute. Because the data are cross‐sectional, the finding describes a difference between age groups, not a change within individuals. Katibi et al. reported normal limits stratified by age and sex in Nigerians (Katibi et al. 2013); we did not identify a formal test of an age‐by‐sex interaction for QRS duration in the Nigerian studies we reviewed, and the present finding should be treated as hypothesis‐generating until replicated in an unselected sample.

Whether an ECG is reported as normal also depends on morphology, and the criteria applied matter in a Black African population. Anterior T‐wave inversion in V1–V4 preceded by J‐point elevation and convex ST‐segment elevation is regarded as a normal variant in Black athletes (Sharma et al. 2018), and applying the 2010 European Society of Cardiology criteria classified 40.4% of ECGs in Black athletes as abnormal, compared with 11.5% using refined criteria (Sheikh et al. 2014). The early repolarization pattern, defined in a consensus statement (Macfarlane et al. 2015), may similarly influence classification. No included ECG had a heart rate below 60 bpm (Section 3.6), so sinus bradycardia, which is common in athletes and regarded as a normal training‐related finding (Sharma et al. 2018), appears to have led to exclusion. How early repolarization, anterior T‐wave inversion and isolated voltage criteria for left ventricular hypertrophy were handled could not be determined, because morphology was not extracted and the reasons for individual classifications were not recorded. If such patterns led to exclusion, the included sample would under‐represent adults with these variants, although patterns described in athletes may not carry the same meaning in a diagnostic‐centre population. Future studies should record these patterns explicitly.

Several unmeasured factors may also have influenced the results. Clinical histories were not reviewed, no data were available on comorbidity, medication, blood pressure or body size, and neither the reason for referral nor habitual physical activity was extracted. Reasons for referral are likely to differ by age and sex, and hypertension and QT‐prolonging medication are more common in older adults, which could contribute to the longer QTc in the older group. Habitual physical activity may also influence sex differences: in adult Rugby Football League athletes, males trained more hours per week than females, and after adjustment for weekly training hours only the sex differences in PR interval and QRS duration remained significant (Warrington et al. 2025). That athletic population differs considerably from the present sample, and sex differences in heart rate and QTc are well established in nonathletes, but physical activity can influence resting heart rate and, through residual rate dependence, rate‐corrected QTc.

Table 7 compares the present values with two published African datasets. QRS and PR values were broadly similar to those reported by Dewhurst et al. in older rural Tanzanians, particularly for the older subgroup, although the Tanzanian values were adjusted for age, systolic blood pressure, body mass index and RR interval and the present values were not (Dewhurst et al. 2014). Present QTc values were higher than those reported by Katibi et al., who used the Hodges formula (Katibi et al. 2013). Because Bazett's formula gives higher QTc values than Hodges' formula at heart rates above 60 bpm (Vandenberk et al. 2016), formula differences could explain much of this gap if the present device used Bazett's formula, but as the formula has not been confirmed, the comparison is indicative only. Awoyinka and Ogunlade reported values for adults of Yoruba ethnicity aged 18–40 years (Awoyinka and Ogunlade 2024); that study also examined sex differences in these parameters, but its values were not included in Table 7 because it was restricted to young adults of a single documented ethnicity. Because ethnicity was not recorded in the present study, these comparisons are between study populations and not between ethnic groups.

Taken together, the present values are in the same range as other African datasets, and the QTc correction method is a major source of uncertainty in cross‐study comparisons.

For clinicians at this and comparable diagnostic centres, the findings support the use of sex‐specific limits when interpreting QTc in adults, in line with established practice. The QRS finding should not change practice until replicated in an unselected sample. For researchers, three priorities follow: (i) documenting the QTc correction formula used at Nigerian diagnostic centres, since this methodological choice may be a larger source of cross‐study disagreement than biological differences between cohorts; (ii) repeating the age comparison, including the age‐by‐sex QRS interaction, in a community‐based or unselected sample that does not prefilter on a normal ECG; and (iii) recording ethnicity, nationality, reason for referral, comorbidity, medication, body size and physical activity.

In summary, the female‐longer QTc was present in both age groups and was robust to adjustment for heart rate and to uniform application of conventional limits, and it is the finding best supported by these data. The age comparisons for PR interval and QRS duration, and the QRS age‐by‐sex interaction may have been affected by selection on a normal ECG. Confirmation of the QTc correction formula remains a priority before these values are used clinically.

## 5 Limitations

1. QTc values were those reported by the ECG machine. The correction formula was not documented in the source records and could not be confirmed retrospectively, and the uncorrected QT and RR intervals were not extracted, so QTc could not be recalculated with alternative formulae such as Fridericia's or Framingham's (Vandenberk et al. 2016). The residual rate dependence of QTc (Section 3.7) does not identify the formula and cross‐study QTc comparisons (Table 7) are indicative only.
2. Only ECGs reported as normal were included, and each was classified by a single cardiologist without independent re‐adjudication. ECG classification is not always clear‐cut, particularly for borderline intervals and repolarization findings, and interpretation varies between readers; the observed data show that conventional limits were not applied uniformly (Section 3.6). Because the classification depends partly on the intervals studied, it limits how much age‐related prolongation of PR interval and QRS duration could be observed. Selection is therefore a possible explanation for the null PR and QRS age findings and for the QRS age‐by‐sex interaction, which was not robust to uniform application of the limits, and the values in Table 5 are conditional on prior normal classification, not population‐representative normal limits.
3. Ethnicity, nationality, place of birth and language were not recorded and cannot be reconstructed. Although the study was designed to address a gap in evidence for Nigerian adults, residence in the catchment area of a centre in Owerri does not establish Nigerian nationality or Igbo ancestry. The findings therefore apply to adults attending a single diagnostic centre in Owerri, and comparisons with cohorts of documented ethnicity, such as the Yoruba cohort (Awoyinka and Ogunlade 2024), cannot be interpreted as ethnic comparisons.
4. The retrospective, single‐centre, purposive‐sampling design limits generalizability to other regions and diagnostic settings and precludes formal random‐sampling‐based inference.
5. No data on reason for referral, body mass index, blood pressure, comorbidity, medication use, habitual physical activity or autonomic state (e.g., recent activity, caffeine intake) were available. These factors may differ by age and sex and can influence heart rate, QRS duration and QTc.
6. The cross‐sectional, two‐age‐band design (18–40 vs. ≥ 60 years, with the 41–59‐year band unsampled) cannot establish within‐individual aging trajectories; the age‐group comparison reflects a between‐cohort contrast, not a longitudinal aging effect, and unmeasured cohort differences between adults now aged 60+ and those now aged 18–40 cannot be excluded. The whole‐sample continuous‐age correlation (Section 3.2) is reported for completeness but is a secondary, less interpretable analysis given the bimodal age distribution this design produces.
7. The study design intentionally excluded parameters relevant to left ventricular hypertrophy and used only corrected QT rather than raw QT and QT dispersion, which limits the scope of repolarization assessment possible from this dataset. P‐wave duration was a global automated measurement, so conventional single‐lead thresholds for P‐wave duration cannot be applied to these values.
8. Formal inter‐rater agreement statistics between the cardiologist's original read and the physiologist's re‐measurement were not computed for this dataset (Section 2.4) and should be reported in future work using this archive.
9. Multiple comparisons were addressed with a Bonferroni‐corrected family‐wise threshold (*α* = 0.01); all findings emphasized in the Abstract and Conclusion survive this correction, but the residual risk of Type I error remains for results reported only at the nominal *α* = 0.05, and these are flagged accordingly in the text.
10. While the achieved sample (*n* = 313) is substantially larger than the number originally proposed, no prospective power calculation was performed, each age‐by‐sex partition was smaller than the 120 individuals recommended for nonparametric reference limits (Clinical and Laboratory Standards Institute 2010), and the age range 41–59 years was not sampled, so these descriptive values should not be extrapolated to that middle‐age band without direct study.

## 6 Conclusion

In adults whose ECGs had been reported as normal at a single diagnostic centre in Owerri, Nigeria, females had a longer QTc than males in both age groups (14.6 ms in younger and 11.6 ms in older adults), and this finding was robust to adjustment for heart rate and to uniform application of conventional limits. Older adults had a modestly longer QTc than younger adults, also after adjustment for heart rate. The male–female difference in QRS duration was smaller in older than in younger adults, but this cross‐sectional finding was not robust and is regarded as hypothesis‐generating. PR interval, QRS duration, P‐wave duration and heart rate did not differ significantly between age groups. Selection on a normal ECG, reflected in truncation of the PR interval at 200 ms in both age groups, is a possible explanation for the null PR and QRS findings, but a true absence of an age‐related difference cannot be excluded. The age‐ and sex‐stratified values in Table 5 are conditional descriptive values, not reference intervals, pending prospective, multicentre validation in an unselected sample with recorded ethnicity and a documented QTc correction formula. These findings support sex‐specific interpretation of QTc in this setting. Confirming the QTc correction formula used at Nigerian diagnostic centres, quantifying inter‐rater agreement, and replicating the age comparisons in an independent, population‐based cohort are priorities for future research.

## Author Contributions

Conceptualization: J.E.E. Methodology: C.N.U., C.N.E., A.G., G.O., I.P. Formal analysis: J.N.O. Data curation: C.N.U., C.N.E., A.G., G.O., I.P. Writing – original draft: J.N.O. Writing – review and editing: C.N.U., C.N.E., A.G., G.O., I.P., J.N.O. Supervision: C.N.U., C.N.E., A.G., G.O., I.P. All authors read and approved the final manuscript.

## Funding

The authors have nothing to report.

## Ethics Statement

Ethical approval was obtained from the School of Basic Medical Sciences Ethics and Research Committee, Federal University of Technology Owerri (FUTO), Nigeria (Approval No. FUT/COM/SBMS/REC/Vol1/002; approved on 27 October 2025), prior to data extraction. This retrospective study utilized fully de‐identified archival electrocardiographic (ECG) data. The requirement for individual informed consent was waived by the approving ethics committee because the study involved no direct participant contact, used only anonymized retrospective records, and posed minimal risk to participants, in accordance with local ethical guidelines for retrospective record‐based research. The study was conducted in accordance with the principles of the Declaration of Helsinki.

## Consent

The authors have nothing to report.

## Conflicts of Interest

The authors declare no conflicts of interest.

## Data Availability

The de‐identified dataset analyzed in this study is available from the corresponding author on reasonable request.
